# Eupafolin ameliorates lipopolysaccharide-induced cardiomyocyte autophagy via PI3K/AKT/mTOR signaling pathway

**DOI:** 10.22038/ijbms.2019.37748.8977

**Published:** 2019-11

**Authors:** Yan Gao, Yi Zhang, Yangyang Fan

**Affiliations:** 1 Function Testing Lab, Shaanxi Provincial People’s Hospital, Xi’an, Shaanxi P.R. China; 2 ICU Department, Shaanxi Provincial People’s Hospital. Xi’an, Shaanxi P.R. China; 3 Obstetrical Department, Shaanxi Provincial People’s Hospital. Xi’an, Shaanxi P.R. China

**Keywords:** Autophagy, Cardiomyocyte, Eupafolin, Lipopolysaccharides, Mammalian target of - rapamycin

## Abstract

**Objective(s):**

Eupafolin, a major active component of *Eupatorium perfoliatum L*., has anti-inflammatory and anti-oxidant properties. Lipopolysaccharide (LPS) is responsible for myocardial depression. A line of evidences revealed that LPS induces autophagy in cardiomyocytes injury. This study aims to evaluate the effects of eupafolin on LPS-induced cardiomyocyte autophagy.

**Materials and Methods:**

The effect of LPS on cell viability was examined by CCK-8. Autophagic protein 2 light chain 3 (LC3II), which was regulated by LPS and eupafolin, was examined using immunofluorescent staining. The expression levels of Beclin-1 and p62 were detected by western blotting. The effects of eupafolin on phosphatidylinositol-3-kinase/ protein kinase B/ mammalian target of rapamycin (PI3K/AKT/mTOR) signaling pathway were also evaluated by western blotting and immunofluorescent staining.

**Results:**

Eupafolin pretreatment reduced the expression of LC3II and Beclin-1, whereas p62 was significant increased. In addition, eupafolin promoted expression of PI3K/AKT/mTOR signaling pathway and mTOR inhibitor rapamycin reversed the inhibitory effects on LPS-induced cardiomyocyte autophagy.

**Conclusion:**

Eupafolin exerts anti-autophagy activity via activation of PI3K/AKT/mTOR signaling pathway.

## Introduction

A variety of heart diseases including cardiac hypertrophy and ischemia reperfusion injury were observed with autophagy activation (1). Autophagy is an intracellular degradation process, which is highly regulated by removing cytosolic long-lived proteins and damaged organelles from cells. Inappropriate stimulation of autophagy may promote cell death, whereas other researches point to offer protective effects for autophagy (2-4). The sepsis-induced myocardial injury is also modeled by lipopolysaccharide (LPS)-induced inflammation and oxidative stress (5). LPS-induced autophagy is a cytoprotective response to suppress programmed cell death (6). However, excessive autophagy can also induce cardiac dysfunction and cell death (7). Recently, it has been found that LPS leads to multiorgan dysfunction and is critical in myocardial dysfunction. LPS is reported to induce mitochondrial biogenesis and autophagy in cardiomyocytes (6). Researches indicate that LPS-induced cardiomyocyte autophagy is a leading cause of death in patients with sepsis. The natural medicinal plants have been investigated as a potential therapeutic strategy for dysfunction of cardiovascular disease via regulating autophagy (8, 9). Thus, the moderation of autophagy is essential to reduce LPS-induced myocardial injury.

Eupafolin is an active component of *Eupatorium perfoliatum L., *which is a traditional herbal medicine from China and India (10, 11). Eupafolin has been investigated to show anti-inflammatory, anti-oxidant and anti-tumor effects (12-14). Various flavonoids with anti-oxidant effects have been revealed to suppress lipid peroxidation and increase the anti-oxidation capacity of myocardial cells (15, 16). Eupafolin is also a flavonoid compound. Recent studies reveal various effects of eupafolin on pathologies including, but not limited, inflammation in acute renal injury, apoptosis in cervical cancer and mitochondrial metabolism (12, 17, 18). However, its pharmacological effects on cardiac disease have been poorly studied. Although previous studies have evaluated some effects of eupafolin, its specific effects on cardiomyocyte autophagy are unclear. 

Eupafolin has been reported to exert anti-inflammation and anti-oxidative stress properties by regulating various signaling pathways, including Jun N-terminal kinase (JNK), nuclear factor- kappa B (NF-κB), mitogen-activated protein kinases (MAPK), and protein kinase B (AKT) pathways (19-21). In addition, cyclooxygenase-2 (COX-2) usually acts in inflammatory response, which has been revealed to be affected by eupafolin (22). Previous research indicated that LPS-induced autophagy is associated with phosphatidylinositol-3-kinase/ AKT/ mammalian target of rapamycin (PI3K/AKT/mTOR) signaling pathway (23, 24). PI3K/AKT/mTOR signaling pathway has been a classical pathway in regulating autophagy. The kinase mTOR includes two mTOR complexes, mTORC1 and mTORC2. mTORC1 is a negative regulator of autophagy (25). In addition, PI3K/AKT pathway is an upstream modulator of mTORC1 (26, 27). A research has shown that LPS-induced autophagy is regulated by AKT/mTOR pathway (28). Beclin-1 is a core component of Class III PI3K complex, which is associated with a variety of cellular processes such as autophagy (29). Researches indicated that mTOR can negatively regulate Beclin-1 expression in many kinds of diseases such as cerebral ischemia reperfusion and hepatocellular carcinoma (30, 31). p62, a classical cargo receptor of autophagy, is an LC3-interacting protein and is constantly degraded by the autophagy-lysosome system (32). 

Limited studies involve the effects of eupafolin on LPS-induced myocardial injury, so we hypothesized that eupafolin could attenuate LPS-induced autophagy through downregulating PI3K/AKT/mTOR signaling pathway. This study provides a theoretical basis for the role of eupafolin in the treatment of myocardial injury.

## Materials and Methods

***Cell culture and treatment***

Human myocardial cell lines HL-1 was cultured in Claycomb medium (sigma), supplemented with 10% fetal bovine serum (FBS; Gibco), 100 U/ml penicillin, 100 μg/ml streptomycin (Gibco), 2 mM L-glutamine (Gibco) and 100 μM noradrenalin (Gibco), and incubated at 37 °C, and 5% CO2. Treatment with rapamycin (0.1 μM), and eupafolin (10, 20, 50 μM) were accomplished by adding these compounds 1 hr before LPS treatment. To establish cardiomyocyte autophagy, cells were treated with LPS (sigma) in concentration of 100 μg/ml or 200 μg/ml for 0, 4, 6, 8, and 16 hr.

***CCK-8***

Cell viability was examined by Cell Count Kit-8 (CCK-8) assay. Cells were seeded at 1.5 × 10^3^ cells per well in 96-well culture plates overnight and treated with LPS 100 μg/ml or 200 μg/ml for 0, 4, 6, 8, and 16 hr at 37 °C in a 5% CO_2_ atmosphere. Subsequently, CCK-8 (Transgen Biotech, Shanghai, China) solution (10% of the medium) was added into each well and incubated for 4 hr. The absorbance was measured at 490 nm. 

***Immunofluorescence***

Green fluorescent protein-microtubule associated protein light chain 3 (GFP-LC3) was transfected into cells using Effectene transfection reagents (Qiagen) according to the user’s instructions. The transfection efficiency was visualized by fluorescence microscopy. GFP-LC3-transfected cells were subsequently incubated with LPS or eupafolin in culture medium for the indicated times. Cells were fixed with 4% paraformaldehyde at room temperature for 10 min. Then, cells were inspected at ×100 magnification.

***Western blot***

Cells were seeded at concentration of 8 × 10^5^ cells in each 6-cm dish overnight. Cells were then treated with LPS or eupafolin for indicated times. The cells were harvested and extracted by radioimmunoprecipitation assay (RIPA) buffer (Sigma) at 4 °C. After 15 min centrifugation at 15000 g, supernatants were collected and stored at -20 °C. Total proteins (40 μg) were resolved by SDS-PAGE, and transferred onto a polyvinylidene difluoride membranes (PVDF) and incubated with the following antibodies: glyceraldehyde-3-phosphate dehydrogenase GAPDH) (1/1000; Cell signaling technology), Beclin-1, p62 (1/1000; Cell signaling technology), phosphor-PI3K (1/1000; Cell signaling technology), PI3K (1/1000; Cell signaling technology), phosphor-AKT (1/1000; Cell signaling technology), AKT (1/1000; Cell signaling technology), phosphor-mTOR (1/1000; Cell signaling technology), mTOR (1/1000; Cell signaling technology). Blots were following incubated with horseradish peroxidase (HRP)-linked goat anti-rabbit IgG antibody for 1 hr at room temperature. The bands were detected using the ECL chemiluminescent substrate reagent kit (Thermo Fisher Scientific). The intensity of each band was finally analyzed using a densitometer. 

***Statistical analysis***

Results were analyzed with Graphpad 6.0. The probability of statistically significant differences was tested by GraphPad Prism 6.0 using one-way ANOVA followed by Turkey’s *post hoc* test for multiple comparisons and students’ t test for comparison between groups. Values were expressed as means ± SD of at least three independent experiments. *P-*value of less than 0.05 was considered statistically significant. 

## Results

***LPS induced autophagy of cardiomyocyte***

**Figure 1:**
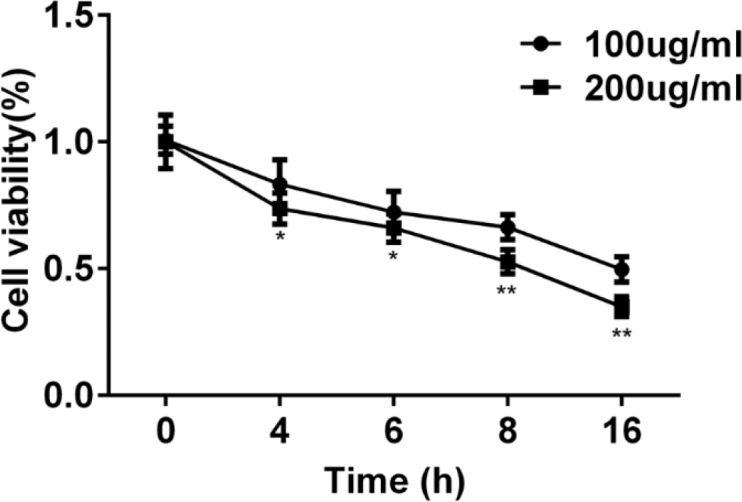
Effects of eupafolin on cell viability. CCK-8 assay was used to detect cell viability of cardiomyocyte after treatment with eupafolin (100 μg/ml or 200 μg/ml). Data were presented with mean±SD, *P<0.05, **P<0.01, versus 100 μg/ml

**Figure 2:**
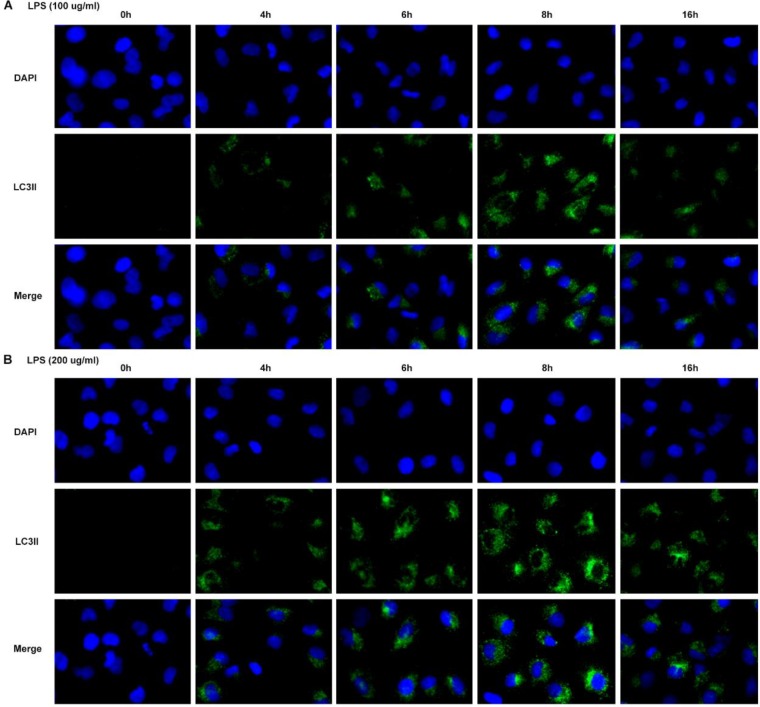
LPS-induced autophagy in cardiomyocyte. A: LPS with 100 μg/ml induced expression of LC3II at 0 hr, 4 hr, 6 hr, 8 hr and 16 hr. B: LPS with 200 μg/ml induced expression of LC3II at 0 hr, 4 hr, 6 hr, 8 hr and 16 hr. Data were expressed as mean±SD, *P<0.05, **P<0.01, ***P<0.001 versus 0 hr. LPS: Lipopolysaccharide, LC3II: Protein 2 light chain 3

**Figure 3:**
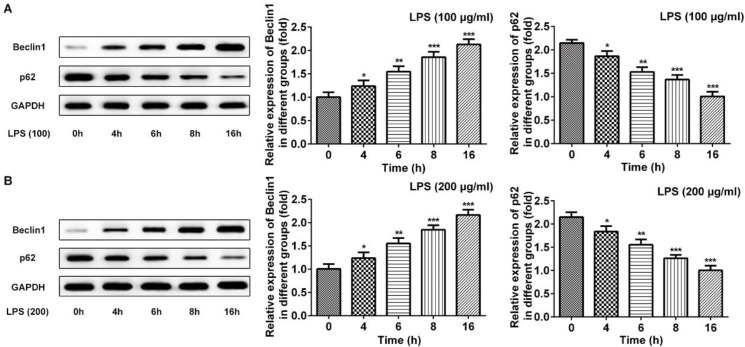
LPS-induced expression of Beclin-1 and p62 in cardiomyocyte. A: LPS with 100 μg/ml induced expression changes of Beclin-1 and p62 at 0 hr, 4 hr, 6 hr, 8 hr and 16 hr. B: LPS with 200 μg/ml induced expression of Beclin-1 and p62 at 0 hr, 4 hr, 6 hr, 8 hr and 16 hr. Data were expressed as mean±SD, *P<0.05, **P<0.01, ***P<0.001 versus 0 hr. LPS: Lipopolysaccharide

To choose the best condition that can induce autophagy of cardiomyocyte, cell viability was first detected. Cell viability was decreased to 50% at condition of LPS 100 μg/ml for 16 hr and LPS 200 μg/ml for 8 hr (Figure 1). Subsequently, protein 2 light chain 3 (LC3II) was significantly increased at 100 μg/ml or 200 μg/ml for 8 hr (Figure 2). The autophagic proteins Beclin-1 and p62 were detected by western blot. Beclin-1 was significantly increased and p62 was decreased significantly at 16 hr both in cells treated with LPS 100 μg/ml or 200 μg/ml (Figure 3). In order to establish a cardiomyocyte autophagy, we chose the condition of LPS 200 μg/ml induced for 8 hr to finish the further study.

***Eupafolin reversed cell autophagy in cardiomyocyte***

**Figure 4:**
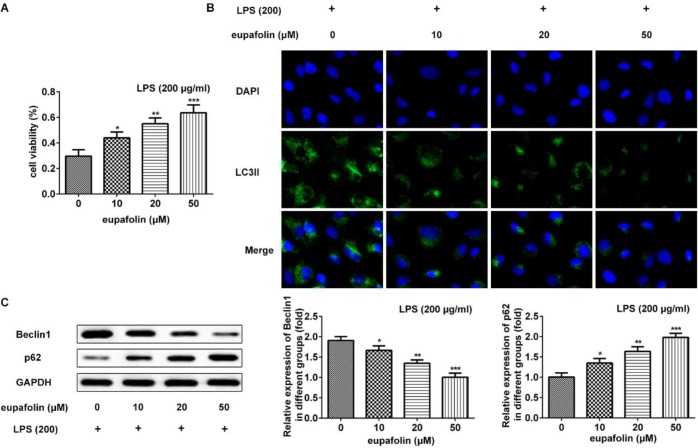
Eupafolin improves LPS-induced autophagy and viability in cardiomyocyte. A: Cell viability of cardiomyocyte was detected after treatment with LPS and eupafolin. B: eupafolin alleviated expression of LC3II in LPS-induced cardiomyocyte. C: Effects of eupafolin on expression of Beclin-1 and p62 in LPS-induced cardiomyocyte. Data were presented with mean±SD. *P<0.05, **P<0.01, ***P<0.001, versus eupafolin 0 μM. LPS: Lipopolysaccharide, LC3II: Protein 2 light chain 3

According to previous investigation (12, 33), three concentration of eupafolin was used to evaluate the effects of eupafolin on LPS-induced autophagy and cell viability. Results showed that eupafolin prevented myocardial injury induced by LPS (Figure 4A). LC3II expression in LPS-induced cardiomyocyte was significantly decreased after treating with eupafolin 50 μM (Figure 4B). Beclin-1 was significantly decreased and p62 was significantly increased after treating with eupafolin 50 μM (Figure 4C). Thus, eupafolin improves LPS-induced autophagy and cell viability in cardiomyocyte. 

***Eupafolin promoted PI3K/AKT/mTOR signaling pathway***

**Figure 5:**
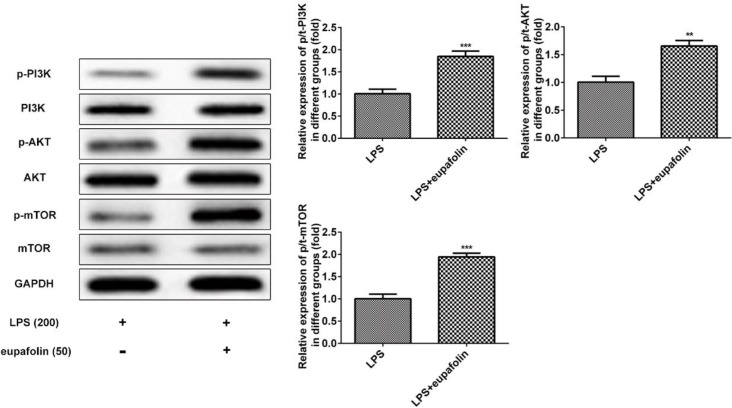
PI3K/AKT/mTOR was activated via eupafolin in LPS-induced cardiomyocyte. Data were presented with mean±SD. **P<0.01, ***P<0.001, versus eupafolin untreated cells. LPS: Lipopolysaccharide; PI3K/AKT/mTOR: Phosphatidylinositol-3-kinase/ protein kinase B/ mammalian target of rapamycin

**Figure 6:**
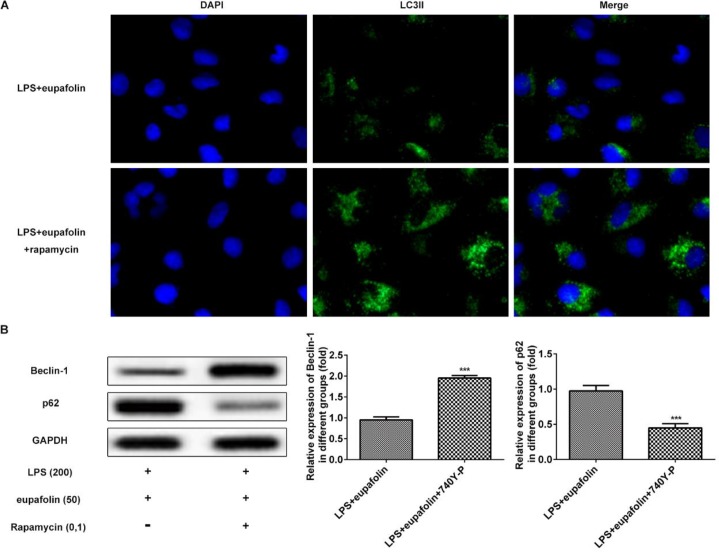
Eupafolin exerts effects on autophagy and viability via PI3K/AKT/mTOR signaling pathway. A: mTOR inhibitor (rapamycin) reversed the effects of eupafolin on expression of LC3II in LPS-induced cardiomyocyte. B: Effects of eupafolin on expression of Beclin-1 and p62 in LPS-induced cardiomyocyte were reversed by mTOR inhibitor. Data were presented with mean±SD. ***P<0.001 versus mTOR untreated group. LPS: Lipopolysaccharide; LC3II: Protein 2 light chain 3; PI3K/AKT/mTOR: Phosphatidylinositol-3-kinase/ protein kinase B/ mammalian target of rapamycin

We further investigated the effects of eupafolin on PI3K/AKT/mTOR signaling pathway. As shown in Figure 5, eupafolin significantly upregulated the expression levels of p-PI3K, p-AKT and p-mTOR in LPS-induced cardiomyocyte. To further investigate whether eupafolin inhibited autophagy in cardiomyocyte via PI3K/AKT/mTOR signaling pathway, rapamycin, an inhibitor of mTOR, was used before eupafolin treatment. Results showed that rapamycin significantly increased LC3II expression even though cardiomyocytes treated with LPS and eupafolin at the same time (Figure 6A). In addition, the expression alterations of Beclin-1 and p62 also proved that rapamycin can reverse the anti-autophagy effect of eupafolin (Figure 6B). These results indicated that eupafolin exerted negative effect on LPS-induced cardiomyocyte autophagy. 

## Discussion

In the present study, we demonstrated that LPS-induced autophagy is associated with excessive autophagy. Eupafolin attenuated the LPS-induced autophagy by activation of PI3K/AKT/mTOR signaling pathway. As per our knowledge, this is the first study to investigate the effects of eupafolin on autophagy in cardiomyocytes exposed to LPS.

Because myocardial injury caused by sepsis is very similar to LPS-induced myocardial injury (5, 34), LPS is often used for myocardial injury modeling. Autophagy exerts cardioprotection by removing toxic oxidative protein (35). However, excessive autophagy would results in autophagic cell death (36). The decrease of age-related autophagy may lead to heart diseases, including LPS-induced myocardial injury (37). In the present *in vitro* study, eupafolin could decrease the LPS-induced autophagy and increase the cardiomyocyte viability. Thus, eupafolin plays a potential role in protecting myocardium by inhibiting myocardial autophagy. 

Autophagy is a conserved process, which involves the degradation and cytosolic components reusing (38). Autophagic initiation is usually controlled by Beclin-1 and LC-3II, which their levels are correlated with the extent of autophagosomes formation. Additionally, p62 is involved in maturation of autophagosomes by selectively incorporating into autophagosomes and subsequently degradation of autolysosomes (38). In this investigation, results showed that LPS exposure promotes autophagic flux by Beclin-1 and LC3II upregulation and p62 degradation. Eupafolin is an important herbal product that exerts anti-inflammatory effects in LPS-induced inflammation (33). Tissue injury promotes a local and systemic inflammatory response without microbial pathogen, a process which induces further injury and a mechanism of disease progression (39). Autophagy may play a permissive role in inflammation observed in the heart during cardiac remodeling (40). In this study, eupafolin inhibited the LPS-induced autophagy by regulating the expression of Beclin-1 and LC3II, thereby reducing the expression of p62. 

A significant body of evidence reveals that PI3K/AKT pathway serves as a critical signaling pathway in cell survival; however, some intriguing reports suggest that this pathway could also promote cell death, especially for necrotic cell death (41). The promotion of eupafolin on PI3K/AKT pathway corresponds to the activation of AKT downstream targets. mTOR is a major downstream target of AKT, and activation of PI3K/AKT/mTOR has been revealed to inhibit autophagy and apoptosis (42). Autophagy involves in complex signaling pathway including the protein kinases mTOR and PI3K (43). In this study, eupafolin activated PI3K/AKT/mTOR signaling pathway and rapamycin, an inhibitor of mTOR, was used to confirm that eupafolin reduced autophagy via inhibiting PI3K/AKT/mTOR signaling pathway. 

## Conclusion

LPS, a Gram-negative bacterial endotoxin, is widely used to induce sepsis via driving aberrant myocardial injury. Our investigation indicated that eupafolin can inhibit LPS-induced autophagic flux in cardiomyocyte *in vitro*, and eupafolin promotes myocardial viability. In addition, the effect of eupafolin on autophagy was mediated by PI3K/AKT/mTOR signaling pathway in cardiomyocyte. However, *in vivo* studies are still required to further prove the protective effects of eupafolin on myocardium. Furthermore, the effects of eupafolin on LPS induced oxidative injury and inflammation, which are associated with autophagy, and also required further investigation.
